# Engineering a Reliable and Convenient SARS-CoV-2 Replicon System for Analysis of Viral RNA Synthesis and Screening of Antiviral Inhibitors

**DOI:** 10.1128/mBio.02754-20

**Published:** 2021-01-19

**Authors:** Yuewen Luo, Fei Yu, Mo Zhou, Yang Liu, Baijin Xia, Xiantao Zhang, Jun Liu, Junsong Zhang, Yingying Du, Rong Li, Liyang Wu, Xu Zhang, Ting Pan, Deyin Guo, Tao Peng, Hui Zhang

**Affiliations:** a Institute of Human Virology, Key Laboratory of Tropical Disease Control of Ministry of Education, Guangdong Engineering Research Center for Antimicrobial Agent and Immunotechnology, Zhongshan School of Medicine, Sun Yat-sen University, Guangzhou, China; b School of Medicine, Sun Yat-sen University, Guangzhou/Shenzhen, China; c Sino-French Hoffmann Institute, Guangzhou Medical University, Guangzhou, Guangdong, China; d Guangdong Provincial People’s Hospital, Guangdong Academy of Medical Sciences, Guangzhou, Guangdong, China; McMaster University

**Keywords:** COVID-19, SARS-CoV-2, safety replicon

## Abstract

The etiologic agent of COVID-19 is highly contagious and has caused a severe global pandemic. Until now, there has been no simple and reliable system available in a lower-biosafety-grade laboratory for SARS-CoV-2 virologic research and inhibitor screening. In this study, we reported a replicon system which consists of four plasmids expressing the required segments of SARS-CoV-2. Our study revealed that the features for viral RNA synthesis and responses to antivirus drugs of the replicon are similar to those of wild-type viruses. Further analysis indicated that ORF6 provided potent in *trans* stimulation of the viral replication. Some viral variations, such as 5′UTR-C241T and ORF8-(T28144C) L84S mutation, also exhibit their different impact upon viral replication. Besides, the screening of clinically used drugs identified that several tyrosine kinase inhibitors and DNA-Top II inhibitors potently inhibit the replicon, as well as authentic SARS-CoV-2 viruses. Collectively, this replicon system provides a biosafety-worry-free platform for studying SARS-CoV-2 virology, monitoring the functional impact of viral mutations, and developing viral inhibitors.

## Abstract (precis)

COVID-19 has caused a severe global pandemic. Until now, there has been no simple and reliable system available in a lower-biosafety-grade laboratory for SARS-CoV-2 virologic research and inhibitor screening.

## INTRODUCTION

The newly emerging severe acute respiratory syndrome coronavirus-2 (SARS-CoV-2), which caused COVID-19 respiratory disease, has infected more than 14 million people and killed more than 0.6 million worldwide up to the middle of July 2020 (1, 2). This pandemic caused social and economic disruption worldwide. So far, no efficient antiviral drugs and vaccines have been developed for SARS-CoV-2. Like other coronaviruses (order *Nidovirales*, family *Coronaviridae*, subfamily *Coronavirinae*), the SARS-CoV-2 virus contains a single plus-strand genome RNA which is 29.9 kb in length. Sixteen nonstructural proteins (nsp1 to nsp16), 4 structural proteins (spike [S], membrane [M], envelope [E], and nucleocapsid [N]), and 6 accessory proteins (3a, 6, 7a, 7b, 8, and 10) have been identified (GenBank accession no. NC_045512.2). Like SARS-CoV, the first two-thirds of the genome encodes 2 overlapping polyproteins, ORF1a and ORF1b, while ORF1b is expressed by −1 ribosomal frameshifting (3). After translation, these two polyproteins are further processed into 16 mature replicase proteins (nsp1 to nsp16) by 2 virus-specific proteinases: a papain-like proteinase encoded by nsp3 and a 3C-like proteinase encoded by nsp5 (4–7). Most of the mature replicase proteins are believed to form a replicon/transcription complex (RTC) that mediates the synthesis of viral genome RNA (replication) and subgenomic mRNAs (transcription) (8).

The 5′ and 3′ termini of coronavirus genomes contain *cis*-acting RNA elements and play pivotal roles in genomic RNA (gRNA) replication and subgenomic RNA (sgRNA) synthesis (9). The subgenomic mRNAs (sgmRNAs) serve as mRNA template for the structural and accessory proteins translation. All positive-strand sgmRNAs have the same 5′ and 3′ coterminus with the viral gRNA. Synthesis of negative-strand sgRNAs is mediated by both leader transcription regulation sequence (TRS-L) sequence located on 5′ untranslated region (UTR) and body TRS (TRS-B) sequence located upstream of open reading frames. During negative-strand sgRNA synthesis, RdRp (nsp12) pauses at any one of the TRS-B sequences and then continues elongation to the next TRS, or it switches to binding the TRS-L located in the 5′ terminus of the genome and elongation to the 3′ terminus (10, 11).

Infectious clones for SARS-CoV-2 and other coronaviruses including mouse hepatitis virus (MHV)-CoV, SARS-CoV, and Middle East respiratory syndrome CoV (MERS-CoV) had been developed through reverse genetic methods (12–16). Although infectious clones were able to produce infectious authentic viruses, the experiment must be conducted in a biosafety level III laboratory. It limits the molecular virology study and high-throughput screening of anti-SARS-CoV-2 inhibitors. Accordingly, the construction of a safety SARS-CoV-2 replicon system as a convenient tool to study fundamental viral processes and to test antiviral drugs is urgently needed.

The construction of a safe and reliable replicon for coronaviruses has been complicated by their large genome size and the existence of gene instability. Many strategies have been provided to overcome these barriers, such as the insertion of full-length coronavirus DNA into the bacterial artificial chromosome (BAC) plasmid (17–19). However, the BAC vector itself contains a large fragment that could lead to indirect or nonspecific gene expressions and unexpected changes in the cell phenotype. Besides, the large BAC vector is more easily degraded during preparation before transfection, and the preparation itself is labor-intensive and time-consuming.

In this study, we constructed a safe and convenient SARS-CoV-2 replicon system consisting of 4 ordinary plasmids and retaining the necessary viral genes and segments for virus RNA synthesis. The luciferase gene was inserted as a reporter under the control of the M protein transcription regulating sequence. We demonstrated that the RNA synthesis of the replicon system is similar to that of the wild-type virus. We have also examined the impacts of all the viral proteins or some of their mutations popularly occurring in epidemic strains on the replicon RNA synthesis. Further, by screening the clinically used drug library, we have identified several compounds capable of potently inhibiting the replicon and further verified their inhibitory effect upon the authentic SARS-CoV-2 viruses.

## RESULTS

### Design of the SARS-CoV-2 replicon system.

**FIG 1:**
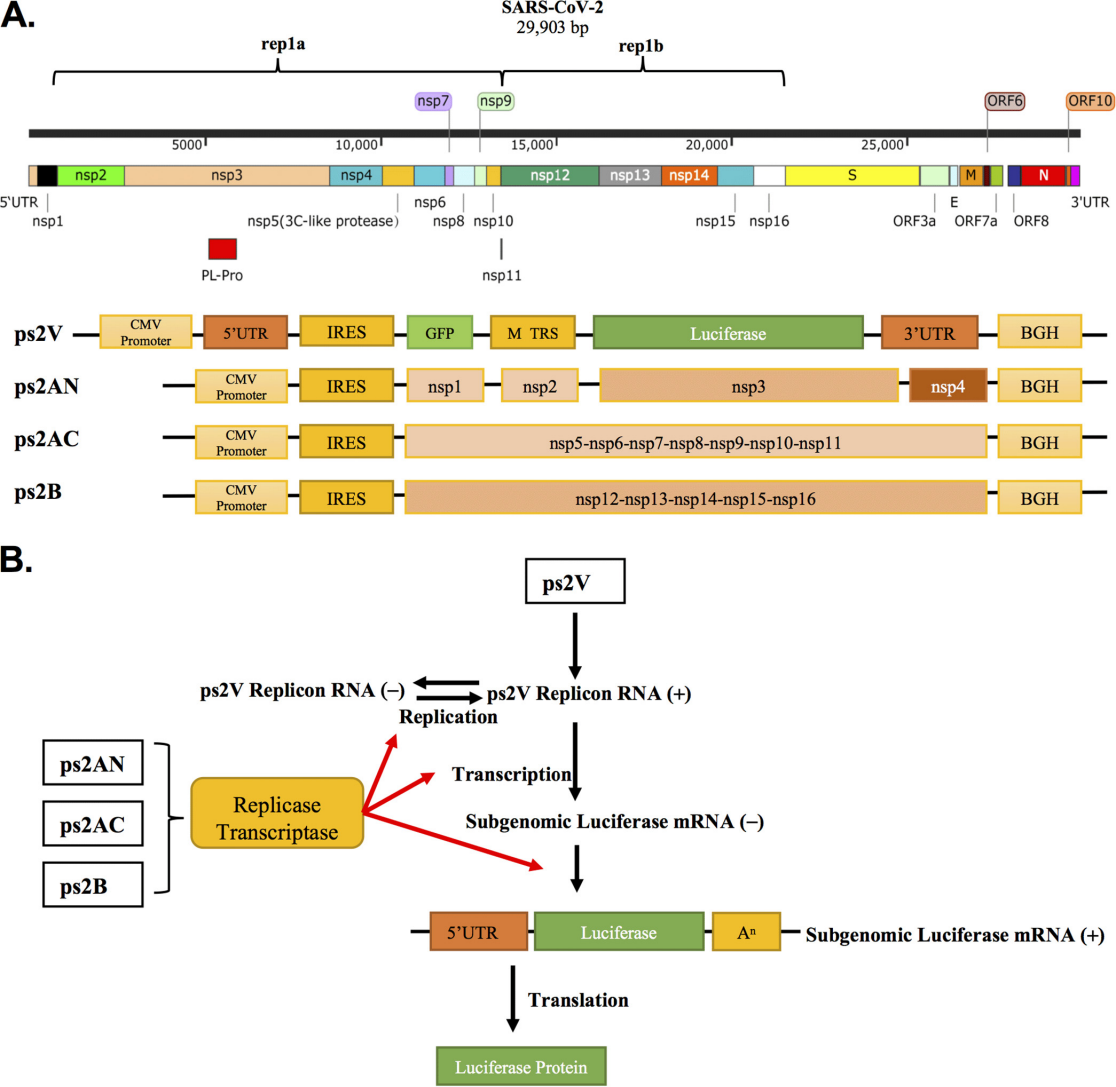
Design of the SARS-CoV-2 replicon system. (A) (Top) The genomic organization of SARS-CoV-2; (bottom) schematic structure of the replicon system consisting of ps2V, ps2AN, ps2AC, and ps2B. (B) The replicases and transcriptases were expressed from ps2AN, ps2AC, and ps2B plasmids. ps2V plasmid expressed the replicon RNA. Replicase and transcriptase mediated replicon replication and the subgenomic luciferase RNA synthesis. The luciferase protein was expressed from subgenomic luciferase RNA.

To simulate the RNA replication and transcription of SARS-CoV-2, a SARS-CoV-2 replicon backbone carrying reporter genes was first constructed. This plasmid retained the 5′ UTR and 3′ UTR of SARS-CoV-2. Between these two segments, the internal ribosome entry site (IRES), which guides the ribosome to start the translation without regular translation initiation such as mRNA capping, was followed by a *gfp* gene with an insertion of quadruple translation termination signal. The green fluorescent protein (GFP) expression could serve as the indicator of efficient transfection and initial RNA transcription under the control of the cytomegalovirus (CMV) or T7 promoter. Importantly, the firefly luciferase gene, a sensitive reporter, was placed downstream of the transcription regulation sequence (TRS; AAACGAAC) of SARS-CoV-2 M proteins but upstream of the 3′ UTR (Fig. 1A). Therefore, the luciferase gene transcription was under the control of the M TRS, which is dependent upon the proper function of mature replicase/transcriptase complex (Fig. 1A).

The self-replication of viral RNA is dependent on the expression and maturation of nsp1 to nsp16 proteins. As the size of nsp1 to nsp16 genes is quite large, we separated them into three constructions: the ps2AN expressing Nsp1 to Nsp4, the ps2AC expressing Nsp5 to Nsp11, and the ps2B expressing Nsp12 to Nsp16. All of them are downstream of an IRES sequence (Fig. 1A). As a result, Nsp1 to Nsp16 were expressed from these three plasmids and formed the mature replicase/transcriptase complex to facilitate replicon RNA replication and transcription (Fig. 1B).

Many virus replicons, including those for Ebola virus, Marburg virus, hepatitis C virus, and dengue virus, were designed with a T7 promoter for driving the initial RNA synthesis (20–23). Alternatively, the BAC-based coronavirus replicon usually used the CMV promoter (17–19). As we did not know which RNA polymerase, bacterial T7 RNA polymerase or host RNA polymerase II, was more suitable for initiating the replicon system, we designed the SARS-CoV-2 replicon system by carrying both the CMV promoter and the T7 promoter. When a T7 DNA polymerase-expressing plasmid was cotransfected with 4 plasmids, we found that GFP expression was significantly decreased (see Fig. S1A in the supplemental material). Therefore, we decided to give up the T7 DNA polymerase driving system and preferred to use the CMV promoter to initiate the SARS-CoV-2 replicon system.

10.1128/mBio.02754-20.1 FIG S1 (A) ps2V (0.1 μg), ps2AN (0.05 μg), ps2AC (0.4 μg), ps2B (0.4 μg), and T7 polymerase expression plasmid (or not) were cotransfected into 293T cells. The GFP signal was determined by flow cytometry. (B to E and I) ps2V (0.1 μg), ps2AN (0.05 μg), ps2AC (0.4 μg), ps2B (0.4 μg), and the indicated constructs or siRNA were cotransfected into 293T cells. The luciferase activity was determined after transfection 30h. (F) The authentic SARS-CoV-2 infection assay was performed according to Materials and Methods. At 48 h postinfection, virus RNA was isolated and RT-qPCR was performed to detect virus RNA copies. (G) Relative Top2b mRNA expression was determined by real-time quantitative PCR after si-Top2B transfection. All above data are representative of three independent experiments. (H) 5′UTR_241C was the dominant strain in the early stage of the epidemic, while the current epidemic strain was mainly 5′UTR_241T. Download FIG S1, TIF file, 0.9 MB. Copyright © 2021 Luo et al. 2021 Luo et al. This content is distributed under the terms of the Creative Commons Attribution 4.0 International license.

### Characterization of the SARS-CoV-2 replicon system.

**FIG 2:**
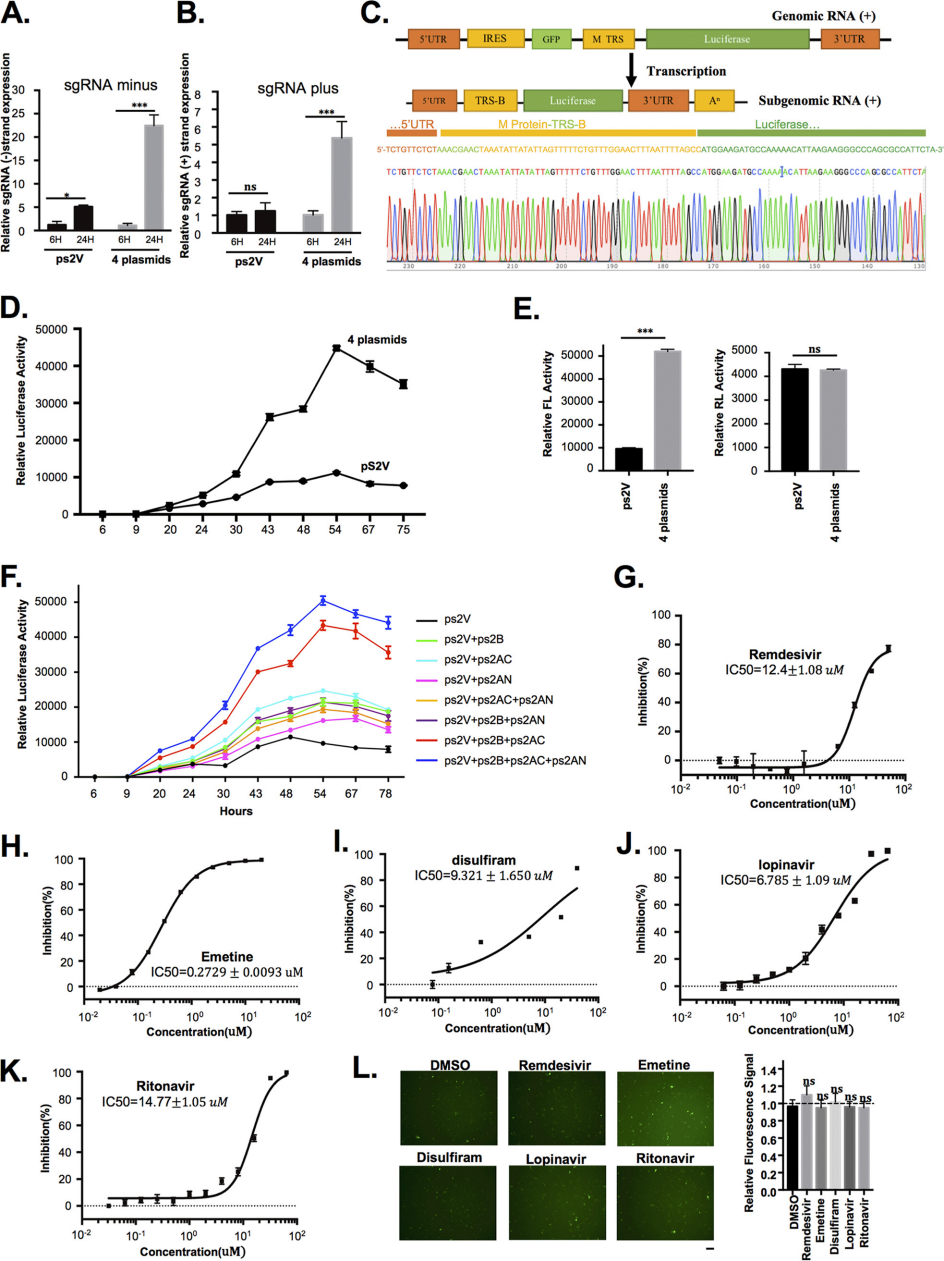
Characterization of the SARS-CoV-2 replicon system. ps2V (0.1 μg), ps2AN (0.05 μg), ps2AC (0.4 μg), and ps2B (0.4 μg) were cotransfected into 293T cells (cell density: 6.5 × 10^4^/cm^2^) seeded in a 12-well plate. (A, B, and C) RNA was isolated 48 h after transfection following treatment with DNase (Promega) before reverse transcription. The RNA was reverse transcribed by specific forward primer for minus RNA or oligo(dT) primer for plus RNA. The minus and plus sgRNA expression was detected through qPCR with specific primers. Human GAPDH mRNA was measured as endogenous controls. The sequence information of primers is listed in Materials and Methods. The PCR products were inserted into T-vector and followed by DNA sequence. (D) The luciferase activity was detected by the luciferase reporter assay system at different time points (hours). (E) The construction expressing renilla luciferase (40 ng) was cotransfected. The luciferase activity was detected after 54 h of transfection by the dual luciferase reporter assay system. FL, firefly luciferase; RL, Renilla luciferase. (F) The mentioned plasmid combinations (consistency of plasmid amount for each group has been ensured via adding empty vector) were transfected into 293T cells. The luciferase activity was detected by the luciferase reporter assay system. (G to K) After 24 to 30 h of transfection, the indicated drugs were added into the cell medium. The luciferase activity was detected after drug treatment for 24 h. IC_50_ of drugs was measured using 9 to 10 different concentrations. The inhibition (%) was calculated by the formula (1 − Luc_each concentration_/Luc_DMSO_) × 100%. (L) The ps2V plasmid was transfected into 293T cells (cell density: 6.5 × 10^4^/cm^2^). After transfection for 12 h, the indicated drugs (10 μM) were added into the cell medium for treatment for 24 h. DMSO treatment served as a negative control. The fluorescence-field micrograph image was obtained and processed in the same way. Scale bar, 100 μm. Images were quantified by Image J software. Graph shows relative fluorescence signal of GFP after different treatments. All experimental data were analyzed using GraphPad Prism. All data are representative of at least three experiments.

To confirm that the luciferase expression of ps2V is derived from the replicon replication/transcription processes but not from nuclear splicing products of the large viral replicon RNA during the CMV promoter-driven transcription in the nucleus, the four plasmids ps2V, ps2AN, ps2AC, and ps2B were cotransfected into 293T cells. The real-time quantitative PCR (RT-qPCR) assay showed a significant increase of minus- and plus-strand sgRNA expression in the 293T cells transfected with the four plasmids compared to that in ps2V-alone-transfected cells (Fig. 2A and B). The existence of subgenomic RNA was further confirmed by sequence analysis (Fig. 2C). Further, the luciferase activity in transfected cells was monitored up to 75 h. It was initially detected at 20 h posttransfection, while the highest value of activity was reached at 54 h posttransfection (Fig. 2D). However, the luciferase activity in ps2V-transfected cells constantly remains at low value. To further verify that the transfection efficiency of the two groups is consistent, we reperformed the experiment via adding the same amount of renilla plasmid into two groups. The result showed that the two groups obtained the same renilla luciferase (RL) activity, but different firefly luciferase (FL) activity, suggesting that the transfection efficiency was consistent (Fig. 2E). To further investigate effects of different combinations of the four plasmids on replicon RNA synthesis, we performed the experiment by separately transfecting different plasmid combinations, such as ps2AN+ps2V, ps2AC+ps2V, ps2B+ps2V, ps2AN+ps2AC+ps2V, ps2AC+ps2B+ps2V, ps2AN+ps2B+ps2V, and ps2AN+ps2AC+ps2B+ps2V. The transfection of ps2V alone served as the control. The result showed that the deletion of either ps2B or ps2AC resulted in a dramatic decrease of luciferase activity, while the deletion of ps2AN had relatively weak effects on luciferase activity, suggesting that the replicon activity depends on the expression of ps2AN, ps2AC, and ps2B, while the expression of ps2AC and ps2B is a prerequisite (Fig. 2F).

To examine whether the replicon could stimulate the wild-type SARS-CoV-2 viruses to respond to the antiviral inhibitors, we examined the system with some of the known anti-SARS-CoV-2 drugs including RdRp inhibitors such as remdesivir or emetine and MLpro inhibitors such as lopinavir, ritonavir, or disulfiram. The concentration inhibiting 50% (IC_50_) of these inhibitors upon replicon RNA synthesis was quantified by detecting luciferase activity: IC_50_ = 12.4 ± 1.08 μM for remdesivir, IC_50_ = 6.785 ± 1.09 μM for lopinavir, IC_50_ = 14.77 ± 1.05 μM for ritonavir, IC_50_ = 0.2729 ± 0.0093 μM for emetine, and IC_50_ = 9.321 ± 1.650 μM for disulfiram (Fig. 2G to K). These results are basically consistent with previous reports (24–27), albeit some controversy remains for remdesivir (28). To further verify whether these drugs have possible effects on replicon plasmid transfection efficiency, the ps2V plasmid alone was transfected into 293T cells. After 12 h of transfection, remdesivir, emetine, disulfiram, lopinavir, and ritonavir were added into cells for 24-h treatment. The result showed that these compounds have little effect on GFP signal, suggesting the inhibition of replicon due to inhibition of viral proteins by these compounds rather than transfection efficiency or others (Fig. 2L). Taken together, these data indicated that the luciferase reporter-based SARS-CoV-2 replicon system could be used as a reliable tool to analyze the viral replication and the inhibitory effect of antiviral compounds targeting the maturation and function of replication/transcription complex.

### Analysis of SARS-CoV-2 protein function based on SARS-CoV-2 replicon system.

**FIG 3:**
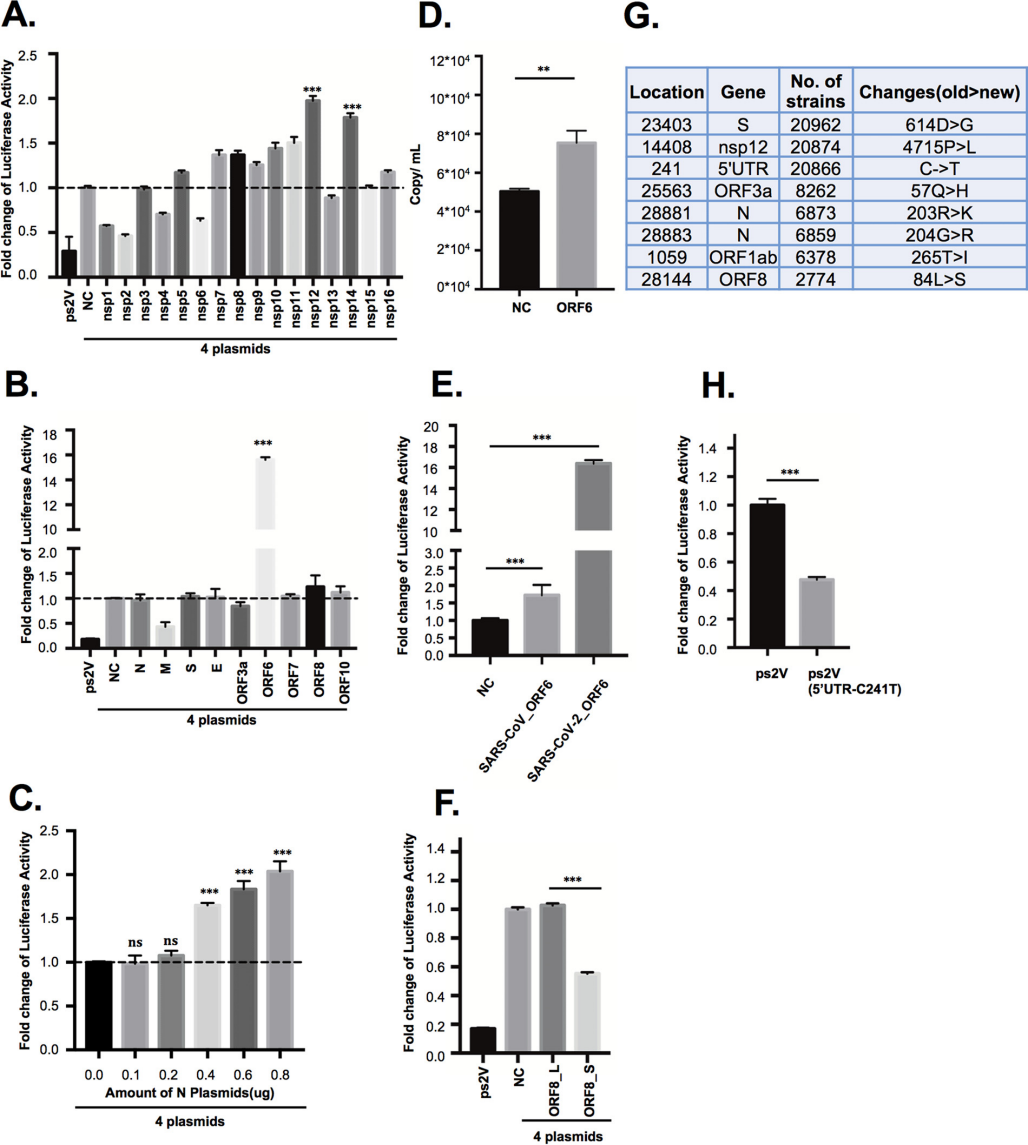
Analysis of SARS-CoV-2 protein function based on the SARS-CoV-2 replicon system. (A, B, E, F, and H) ps2V (0.1 μg), ps2AN (0.05 μg), ps2AC (0.4 μg), ps2B (0.4 μg), and the respective mentioned plasmids (0.2 μg) were cotransfected into 293T cells (cell density: 6.5 × 10^4^/cm^2^) seeded in a 12-well plate. The luciferase activity was detected by the luciferase reporter assay system after 48 to 54 h of transfection. (C) The gradient amounts of N-HA-expressing plasmid (consistency of plasmid amount of each group has been ensured by adding empty vector) were cotransfected into 293T cells with the combination of ps2V (0.1 μg), ps2AN (0.05 μg), ps2AC (0.4 μg), and ps2B (0.4 μg) plasmids. The luciferase activity was detected by the luciferase reporter assay system. (D) The authentic SARS-CoV-2 infection assay was performed according to Materials and Methods. At 48 h postinfection, virus RNA was isolated and RT-qPCR was performed to detect virus RNA copies. (G) The top 8 genome variations with high frequency based on the genome variation information obtained from the high-quality genome sequences of SARS-COV-2 collected from the NCBI and GISAID databases up to the date of 1 July 2020. The strain Wuhan-Hu-1 was retrieved from NCBI (NC_045512.2) as reference for variation annotation. The virus number with the variation is shown in the third column. All experimental data were analyzed using GraphPad Prism. All data are representative of at least three experiments.

To study the effect of all the viral proteins (nsp1 to nsp16, N, S, E, M, ORF3a, ORF6, ORF7, ORF8, and ORF10) encoded by SARS-CoV-2 on replication and transcription, we constructed the plasmids expressing these viral proteins and cotransfected with ps2AN, ps2AC, ps2B, and ps2V, respectively. The expression of individual viral proteins had been confirmed via Western blot analysis (Fig. S2A). Although most of the nonstructural proteins did not exert a significant impact on the replication and transcription of the SARS-CoV-2 replicon, the overexpression of RNA polymerase (Nsp12) or 3′ to 5′ exonuclease (Exon) Nsp14 increased luciferase activity (Fig. 3A). The N protein of coronavirus was reported to increase the rescue efficiency of coronaviruses from infectious RNA transcripts and was required for efficient genome replication (29, 30). For SARS-CoV-2, a recent study indicated that the N protein anatomized IFN-I signaling and efficiently enhanced the replication of virus via interacting with STAT1/STAT2 to inhibit their phosphorylation and nuclear translocation (31). To further investigate whether the N protein promotes the synthesis of replicon RNA, we performed the experiment by transfecting gradient amounts of the N-expressing plasmid with the 4 replicon plasmids. The result showed that a larger amount of N protein enhanced the replicon luciferase activity, suggesting that the effect of nucleocapsid N protein on promoting SARS-CoV-2 genome replication and transcription can be reproduced in our replicon system (Fig. 3C).

10.1128/mBio.02754-20.2 FIG S2 (A) Protein expression of SARS-CoV-2 genes. (Above) High- and medium-molecular-weight protein. (Below) Low-molecular-weight protein. nsp1 to nsp16, ORF3a, ORF6, ORF7, ORF8_S, and ORF8_L, N, S, E, and M with HA tag plasmids (1.5 μg) were separately transfected into 293T cells. nsp11 has only 16 amino acids, while ORF10 has only 38 amino acids; thus, the two proteins are very difficult to blot through Western blotting (WB) (also see in X. Lei, X. Dong, R. Ma, W. Wang, et al., Nat Commun 11:3810, 2020, https://doi.org/10.1038/s41467-020-17665-9). The Western blot assay was performed after 48 h of transfection. β-Actin or GAPDH was used as a loading control. It is difficult to present all the blots on one membrane since the molecular weight of these proteins varies greatly and the exposure time is different due to various expression levels of viral proteins. Therefore, the data were displayed in a discrete way. (B) The effects of drugs on cell viability were detected by CCK8 assay. The 50% cytotoxic concentration (CC_50_) of inhibitors was quantified by detecting OD_450_ absorbance. The data were analyzed using GraphPad Prism. All data are representative of at least three independent experiments. Download FIG S2, TIF file, 0.7 MB. Copyright © 2021 Luo et al. 2021 Luo et al. This content is distributed under the terms of the Creative Commons Attribution 4.0 International license.

Among the structural and accessory viral proteins, ORF6 exerted a more than 10-fold enhancement capability for the replicon system (Fig. 3B). Besides, the ORF6 overexpression also increased the replication of authentic SARS-CoV-2 viruses (Fig. 3D). These data indicated that ORF6 may function as a *trans* factor to promote SARS-CoV-2 replication and transcription. However, the ORF6 of SARS-CoV only slightly increased the replicon RNA synthesis (Fig. 3E). Given that ORF6 of SARS-CoV may block the interferon-α/β (IFN-α/β) signal pathway (32, 33), we knocked down the expression of STAT1 knockdown with small interfering RNA (siRNA). As STAT1 knockdown only mildly enhanced replicon activity and enhanced the effect of ORF6 upon replicon activity (Fig. S1I), the effect of ORF6 of SARS-CoV-2 upon replicon activity is unlikely due to the inhibition of the interferon-α/β (IFN-α/β) signal pathway.

According to the different amino acids (L or S) at position 84 of ORF8, SARS-CoV-2 has been divided into two viral strains: ORF8_84S and ORF8_84L (34–36). Our group previously identified that ORF8 in SARS-CoV-2 potentially downregulated major histocompatibility complex class I (MHC-I) in infected cells and mediated virus immune evasion (37). Although our previous analysis indicated that 84L and 84S subtypes of SARS-CoV-2 ORF8 exerted a similar effect on downregulating MHC-I, we found that ORF8_84L rather than ORF8_84S overexpression showed stronger replicon activity (Fig. 3F), suggesting that the ORF8_84L viral strain is highly likely more virulent.

We have also examined the effect of the 8 most frequent mutations in the epidemic strains upon the replicon (Fig. 3G). Interestingly, the 5′UTR_214C on ps2V showed better replicon activity than ps2V_5′UTR_214T (Fig. 3H). This variation may allow the virus to alternate its replication. However, the D614G at S protein, the P4715L at Nsp12, the Q57H at ORF3a, and the R203K and G204R at N protein have no significant effect upon replicon function (Fig. S1B and C). Collectively, these results indicated that the replicon system could be used as a convenient system for examining the impacts of all the individual viral proteins or some of their mutations commonly occurring in epidemic strains on viral RNA synthesis.

### Clinically used compound library screening based on the SARS-CoV-2 replicon system.

**FIG 4:**
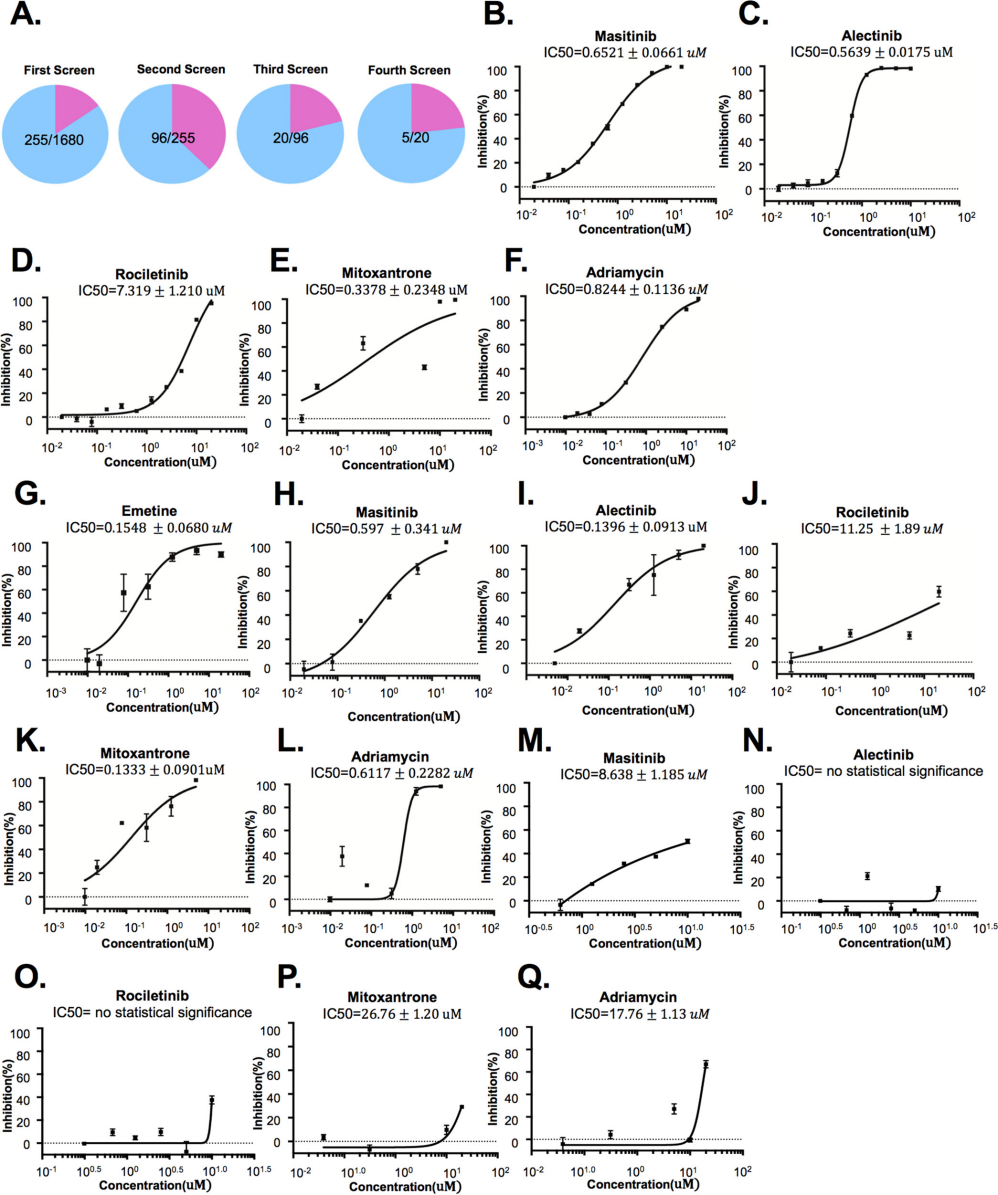
Clinical-use compound library screening based on SARS-CoV-2 replicon system. (A) ps2V (12.5 ng), ps2AN (6.25 ng), ps2AC (50 ng), and ps2B (50 ng) were cotransfected into 293T cells (cell density: 6.5 × 10^4^/cm^2^) seeded in a 96-well plate. After transfection for 24 to 30 h, drugs were added into cell medium for 24-h treatment before detecting luciferase activity. After the fourth round of screening, 5 hit compounds were selected for further examination. (B to F) After transfection for 24 to 30 h, the indicated drugs were added into the cell medium. The luciferase activity was detected after drug treatment for 24 h. IC_50_ of candidate drugs inhibiting replicon was measured using 6 to 10 different concentrations. The inhibition (%) was calculated by the formula (1 − Luc_each concentration_/Luc_DMSO_) × 100%. (G to L) The IC_50_ of candidate drugs inhibiting authentic virus strains. The authentic SARS-CoV-2 infection assay was performed according to Materials and Methods. Drugs were added into cell medium for treatment for 24 h. Virus RNA was isolated, and RT-qPCR was performed to detect virus RNA copies. The inhibition (%) was calculated as (1 − viral RNA copies_each concentration_/viral RNA copies_DMSO_) × 100%. (M to Q) IRES-CMV-luciferase plasmid was transfected into 293T cells (cell density: 6.5 × 10^4^/cm^2^). After transfection for 24 to 30 h, the indicated drugs were added into the cell medium. The luciferase activity was detected after drug treatment for 24 h. IC_50_ of the drugs was measured using different concentrations. The inhibition (%) was calculated by the formula (1 − Luc_each concentration_/Luc_DMSO_) × 100%. All experimental data were analyzed using GraphPad Prism. All data are representative of at least three experiments.

A clinically approved compound library containing 1,680+ drugs was used to screen SARS-CoV-2 inhibitors based on the replicon system (Fig. 4A). Many of the hit drugs such as emetine, ouabain, and digitoxin have been reported to inhibit SARS-CoV-2 (Fig. 4A and Table S1) (26, 38). After 4 rounds of screening and verifications, we selected 5 candidate drugs including masitinib, alectinib, rociletinib, mitoxantrone, and Adriamycin for further study. These selected drugs can be mainly divided into two categories: receptor tyrosine kinase inhibitor and DNA topoisomerase II inhibitor. The IC_50_ upon the replicon was determined: masitinib (0.65210 ± 0.0661 μM), alectinib (0.5639 ± 0.0175 μM), rociletinib (7.319 ± 1.210 μM), mitoxantrone (0.3378 ± 0.2348 μM), and Adriamycin (0.8244 ± 0.1136 μM) (Fig. 4B to F). Effects of drugs on cell viability were determined through CCK8 assay (Fig. S2B). Besides, the inhibitory effects of alectinib, masitinib, rociletinib, mitoxantrone, and Adriamycin on the authentic viruses were determined by measuring IC_50_ with quantification of viral RNA copies. The reported drug emetine was set as a positive reference. Results showed that emetine (IC_50_ 0.1548 ± 0.0680 μM), masitinib (IC_50_ 0.597 ± 0.341 μM), alectinib (IC_50_ 0.1396 ± 0.0913 μM), mitoxantrone (IC_50_ 0.1333 ± 0.0901 μM), and Adriamycin (IC_50_ 0.6117 ± 0.2282 μM) inhibited replication of SARS-CoV-2 (Fig. 4G to L). Rociletinib could potentially inhibit SARS-CoV-2 at a higher concentration (IC_50_ 11.25 ± 1.89 μM). To exclude the possible effects of these drugs on IRES or CMV activity, we have constructed an IRES-CMV-luciferase plasmid. The effects of lomerizine, masitinib, alectinib, rociletinib, mitoxantrone, and Adriamycin on IRES or CMV were assessed by detecting luciferase activity after the IRES-CMV-luciferase plasmid transfection. The result showed that both alectinib (IC_50_ of no statistical significance) and rociletinib (IC_50_ of no statistical significance) have no effects to luciferase activity, while higher concentration of masitinib (IC_50_ = 8.638 μM, 13.2 times IC_50_ of replicon), Adriamycin (IC_50_ = 17.76 μM, 21.5 times IC_50_ of replicon), and mitoxantrone (IC_50_ = 27.76 μM, 82.2 times IC_50_ of replicon) have a slight effect on IRES-CMV-luciferase activity (Fig. 4M to Q). These results suggested that the inhibitions by these drugs of the replicon should be mainly due to their effect on replication-related viral or host factors.

10.1128/mBio.02754-20.3 TABLE S1 Partial inhibition data of drug screening via replicon system. Download Table S1, TIF file, 2.1 MB. Copyright © 2021 Luo et al. 2021 Luo et al. This content is distributed under the terms of the Creative Commons Attribution 4.0 International license.

Collectively, these results indicated that the replicon system can be used as a reliable system for SARS-CoV-2 inhibitor screening. Alternatively, these results also imply that receptor tyrosine kinase pathways and DNA topoisomerase II may influence SARS-CoV-2 replication. By depletion of Top2 A and B with siRNA, we found that both the replicon function and the replication of authentic viruses were inhibited when Top 2B was knocked down (Fig. S1D, F, and G). Conversely, Top 2B overexpression could increase replicon RNA synthesis (Fig. S1E). These data suggest that DNA topoisomerase II-B directly participates in the viral replication.

## DISCUSSION

In this report, we have reported a reliable and convenient SARS-CoV-2 replicon system. As the reporter firefly luciferase gene was under the control of the M protein TRS, it can be expressed only from subgenomic RNAs but not from the replicon genome RNA. Accordingly, the luciferase expression levels could in principle be due to changes in the extent of the replication or the transcription, or both, which are performed by many viral proteins including two viral proteases and RdRp. Since this system does not cover the assembly, budding, and entrance into the viral life cycle, it could, to a large extent, be applied in the study of viral RNA synthesis and drug development without a biosafety level III laboratory.

It has been reported that nsp3, nsp4, and nsp6 induce the formation of double-membrane vesicles (DMV) that are dedicated to viral RNA synthesis (39). In the replicon system, nsp3 and nsp4 were expressed by the ps2AN plasmid, while nsp6 was expressed by the ps2AC plasmid. Figure 2F showed that the lack of ps2AN (nsp1 to nsp4) plasmid has little effect on the luciferase activity of the replicon. There are two possible explanations: (i) the influence of DMV on the replicon activity is more indirect than that of replicase and transcriptase expression and (ii) although a compound named K22 was reported to inhibit viral RNA synthesis by specially targeting DMV, the mutants in nsp6 were resistant to K22, suggesting that nsp6 had a pivotal role in DMV formation during coronaviral infection (40). In addition, the overexpression of nsp6 alone is able to induce the formation of single-membrane vesicles around microtubule organizing centers or endoplasmic reticulum (ER)-derived vesicles which may replace the function of DVM to provide a microenvironment for replicon RNA synthesis (39, 41). We reasoned that despite the lack of ps2AN leading to absence of nsp3 and nsp4 expression, it had little influence on the replicon RNA synthesis due to the presence of nsp6. However, it is notable that our replicon system may not be able to perfectly simulate the formation of DMV structure, so one needs to be cautious when using this system to screen DMV-targeting drugs.

Using the replicon system, we first identified that Nsp14 and ORF6 are beneficial to SARS-CoV-2 RNA synthesis. Previous study in SARS-CoV revealed that Nsp14 usually forms a complex with Nsp10 and functions for RNA viral proofreading (42); our data indicate that a large amount of Nsp10 in viral RNA syntheses is required. Although ORF6 of SARS-CoV may block the interferon-α/β (IFN-α/β) signal pathway through preventing the nuclear import of ISGF3 (Stat1-Stat2-IRF9) complex (32, 33), our study showed that overexpression of ORF6 of SARS-CoV slightly increased replicon RNA synthesis. Besides, we found that STAT1 knockdown mildly enhanced replicon activity and enhanced the effect of ORF6 of SARS-CoV-2. Therefore, its function in viral RNA synthesis is not through the inhibition of the IFN signal pathway and remains to be further determined. Nevertheless, as viral RNA synthesis in SARS-CoV-2 is more sensitive to Nsp14 and ORF6 expression than that of other viral proteins, we reasoned that Nsp14 and ORF6 could be the targets for developing SARS-CoV-2 inhibitors.

During the pandemic of SARS-CoV-2 infection, the frequent mutations of the viral genome could affect the transmissibility and infectivity of the virus. For example, D614G in S protein of SARS-CoV-2 increases the ability of S to bind to ACE2, thereby increasing viral infectivity (43). In this study, we examined the effects of mutations in SARS-CoV-2 epidemic strains upon the replicon and identified that the C241T mutation in the 5′ UTR negatively affects the viral RNA synthesis. The analysis of viral evolution showed that 5′UTR_241C was the dominant strain in the early stage of the epidemic, while the current epidemic strain was mainly 5′UTR_241T (see Fig. S1H in the supplemental material). In this study, our results suggest that the 5′UTR_241C strain may increase virus pathogenicity.

Through drug screening based on the replicon system, we identified 3 receptor tyrosine kinase inhibitors including masitinib, alectinib, and rociletinib as potentially inhibiting SARS-CoV-2 RNA synthesis. A very recent phosphorylation proteomics study revealed that phosphorylation signaling represented a primary host response to SARS-CoV-2 infection (44). Our findings supported that the RNA synthesis of SARS-CoV-2 could be directly dependent on a kind of phosphorylation regulation pathway or indirectly dependent on certain receptor tyrosine kinase pathways. Alectinib (a second-generation anaplastic lymphoma kinase [ALK]-targeted drug) and rociletinib (a third-generation epidermal growth factor receptor [EGFR]-targeted drug), which were used in the treatment of non-small-cell lung cancer with fewer side effects, may be used in animal experiments and clinical trials to further verify the treatment efficacy for COVID-19. In addition, the DNA topoisomerase II inhibitor mitoxantrone and Adriamycin were proved to inhibit SARS-CoV-2 replication. As DNA topoisomerase II-B knockdown and overexpression influenced viral RNA synthesis, it could directly affect the viral RNA replication. Molecular mechanisms underlying this phenomenon remain to be clarified.

We provided a reliable virus replication simulation system to preliminarily assess and analyze the effects of various SARS-CoV-2 genes, the effects of pandemic SARS-CoV-2 gene variants, and the antiviral activities of small compounds. However, the replicon is dependent on an artificial transient-transfection-based system. More authentic viral assay or animal experiments need to be performed to reconfirm these effects.

In summary, we have constructed a safe, convenient, and reliable SARS-CoV-2 replicon system, which has exhibited a powerful capability for virologic study, epidemic monitoring, and antiviral inhibitor screening. Given that we still need to further clarify the molecular mechanism underlying COVID-19 pathogenesis and identify more effective drug targets and subsequently more effective viral inhibitors, this convenient system should be an important tool for us to fight against COVID-19.

## MATERIALS AND METHODS

### Plasmid preparation.

The sequence of the SARS-CoV-2 reference was from isolate SARS-CoV-2 Wuhan-Hu-1 (GenBank accession no. NC_045512.2). The nucleotide sequencing of mentioned plasmids was performed by human codon optimization and chemically synthesized by Genewiz (Suzhou, China) and GenScript (Nanjing, China). The sequence was inserted into pcDNA3.1 plasmid. To ensure sequence stability, TOP10 and Stbl3 were used as competent bacteria. The sequences of indicated plasmids are shown in the supplemental material.

### Cells.

HEK293T cells were obtained from ATCC and maintained in Dulbecco modified Eagle medium (DMEM) supplemented with 10% fetal bovine serum (FBS) (Life Technologies) plus 1% penicillin-streptomycin (Life Technologies) at 37°C with 5% CO_2_. The cells were confirmed to be mycoplasma free before the experiment.

### Plasmid transfection and luciferase reporter assay.

Plasmids or chemically synthesized small interfering RNAs (siRNAs) (siSTAT1, genOFF h-STAT1_1999A_SIGS0000954-1; siTOP2A, si-h-TOP2A_101; standard, 5nmol_ siG000007153A-1-5; siTOP2B, genOFF st-h-TOP2B_001, 5nmol_stB0004038A-1-5; RiboBio, Guangzhou, China) were transfected into 293T cells using Lipofectamine 2000 (Invitrogen) according to the manufacturer’s protocol. Cells were collected and lysed with passive lysis buffer (Promega). The luciferase activity of cell extracts was measured through the luciferase reporter assay system (Perkin Elmer, United Kingdom) according to the manufacturer’s instructions.

### RT-qPCR.

RNA was isolated with TRIzol according to the manufacturer’s instructions (Ambion). The samples were treated with DNase (Promega) before reverse transcription. Total cellular mRNA was reverse transcribed using a specific reverse primer (CAAACCAACCAACTTTCGATCTC) to reverse transcribe minus subgenomic RNA or oligo(dT)s to reverse transcribe plus subgenomic RNA with HiScript II Q select RT supermix (Vazyme). Real-time quantitative PCR was performed to detect minus or plus subgenomic RNA using specific primers (F, CTGTTCTCTAAACGAACTAAATATTATATTA; R, CTCGAAGTACTCGGCATAGGTGA) with the SYBR premix *Ex Taq* kit (TaKaRa) on a CFX96 real-time system (Bio-Rad). Human glyceraldehyde-3-phosphate dehydrogenase (GAPDH) mRNA was measured as endogenous controls.

### High-throughput drug screening and IC_50_ measurement.

The Approved Drug Screening Library contains 1,680 approved drug compounds supplied as predissolved dimethyl sulfoxide (DMSO) solutions with a concentration of 2 mM, which were purchased from TargetMol. All compounds in the library are approved for clinical use by the U.S. Food and Drug Administration (FDA), the European Medicines Agency (EMA), or the China Food and Drug Administration (CFDA). In a screening assay, compounds were added into the medium with a final concentration of 20 μM, with DMSO as a control. Remdesivir, favipiravir, lopinavir, ritonavir, emetine, and disulfiram were purchased from the Selleck Company. Luciferase reporter assay was performed after 24-h drug treatment. IC_50_ of drugs was measured using 9 to 10 different concentrations. All experimental data were analyzed using GraphPad Prism. All experiments were performed in triplicate.

### Infection with authentic SARS-CoV-2.

A SARS-CoV-2 strain named nCoV-19/CHN/SYSU-IHV/2020 strain (accession ID on GISAID: EPI_ISL_444969) was recently isolated by our lab from a female who was infected at Guangzhou by a traveler from Africa in April 2020. For the infection experiment, HEK293T cells (1.6 × 10^5^ cells/ml) were transfected with pCMV-ACE2-Flag and the abovementioned plasmid or siRNA. After 24 h, cells were washed with phosphate-buffered saline (PBS) and infected with authentic SARS-CoV-2 at a multiplicity of infection (MOI) of 0.1 for 1 h at 37°C. Then, cells were washed with PBS and replaced with DMEM (2% FBS) containing various concentrations of the indicated drug compounds. Copy number of SARS-CoV-2 viral RNA was determined through the novel coronavirus (2019-nCoV) nucleic acid diagnostic kit (PCR-Fluorescence Probing) (Daan Gene) according to the manufacturer’s protocol.

### Sequence collection and variation annotation.

The high-quality genome sequences of SARS-COV-2 were collected from the NCBI (https://www.ncbi.nlm.nih.gov/sars-cov-2) and GISAID (https://platform.gisaid.org) databases up to the date of 1 July 2020. The genome sequences were aligned using the MUSCLE program, and the variation annotation results were obtained using VEP software. The genome sequence of the strain Wuhan-Hu-1 was retrieved from NCBI (accession number NC_045512.2) as reference for variation annotation.

### Statistical analysis.

Unless otherwise indicated, values are reported as the mean ± standard error of the mean (SEM); values of *P* < 0.05 were considered significant. Statistical significance between two samples was determined by two-tailed Student *t* test. *P* values are denoted in figures as follows: *, *P* < 0.05; **, *P* < 0.01; and ***, *P* < 0.001.
