# Supplementary figures and images for: Engineering a Reliable and Convenient SARS-CoV-2 Replicon System for Analysis of Viral RNA Synthesis and Screening of Antiviral Inhibitors
Source: mBio. 2021 Jan 19;12(1):e02754-20. doi: 10.1128/mBio.02754-20 (PMC7845634; doi:10.1128/mBio.02754-20)

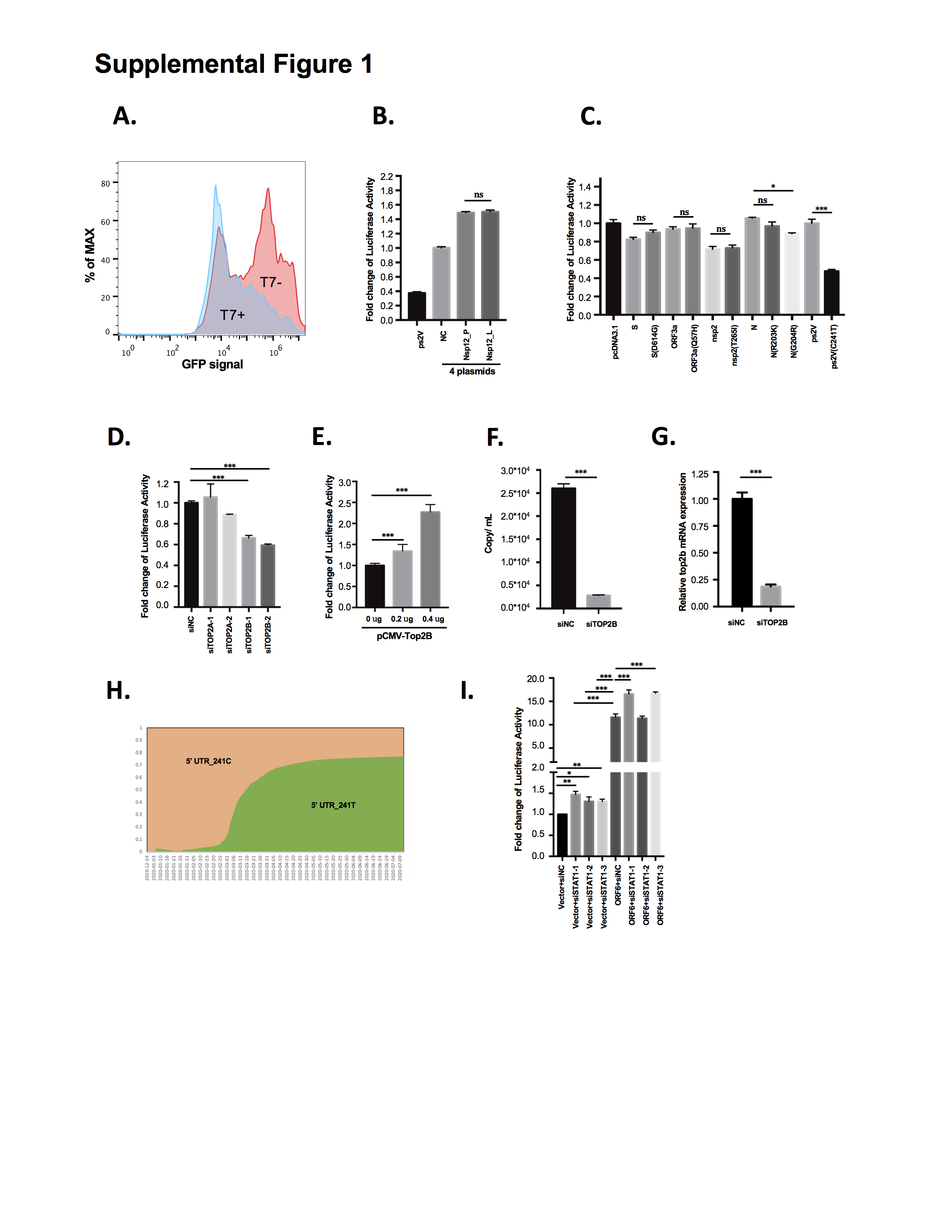

Supplement: FIG S1 [file mBio.02754-20-sf001.tif]

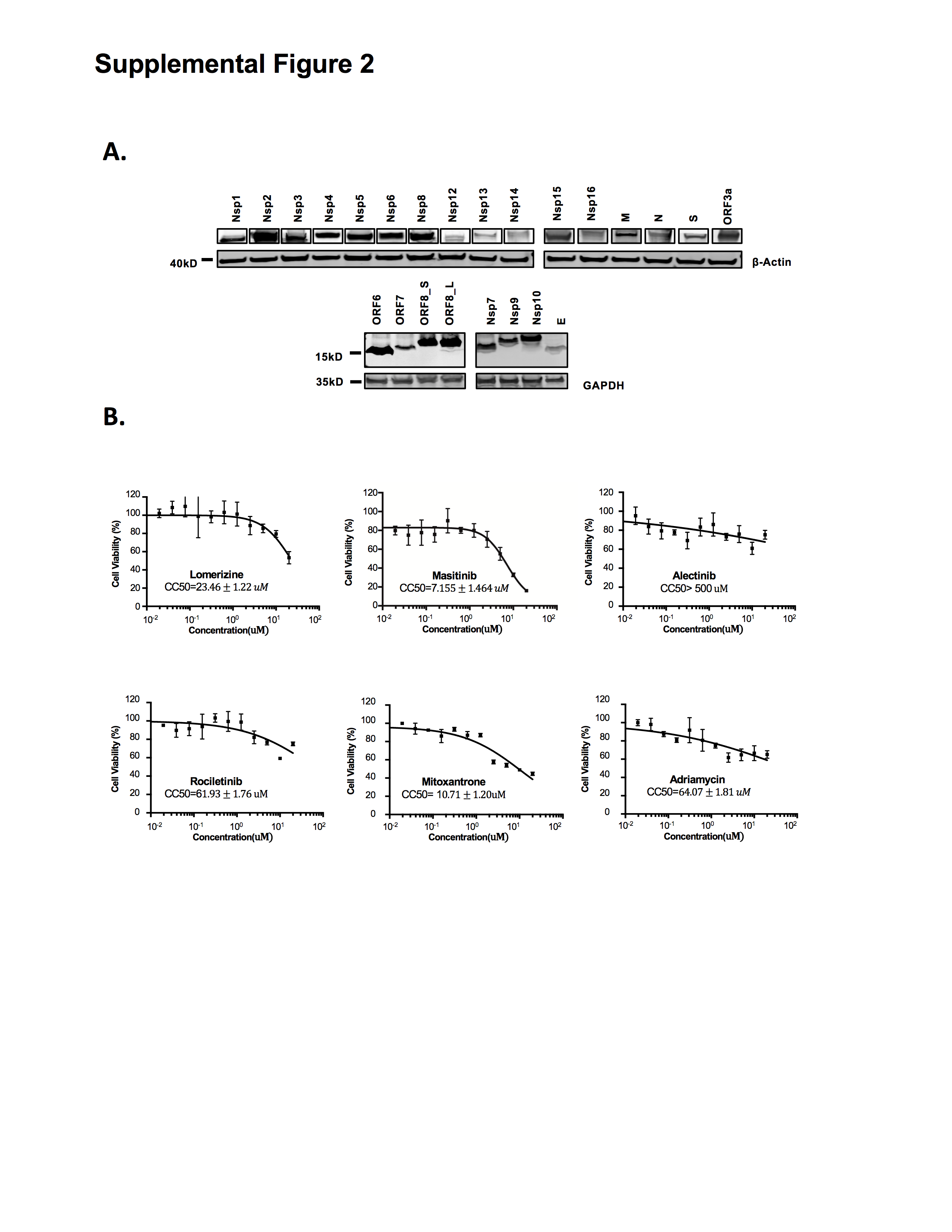

Supplement: FIG S2 [file mBio.02754-20-sf002.tif]

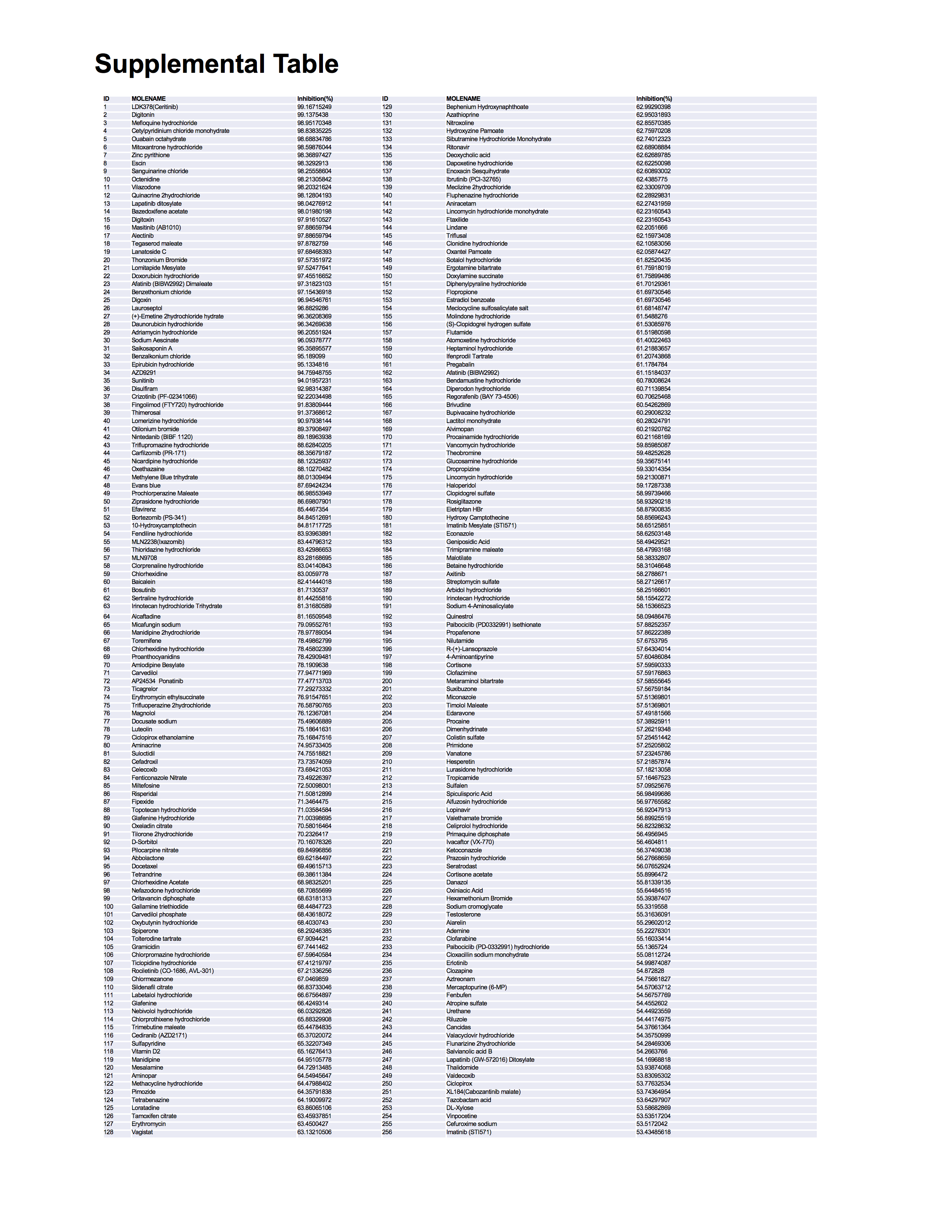

Supplement: TABLE S1 [file mBio.02754-20-st001.tif]
